# The Tiny Epigenetic Addition Plays Big Roles: The RNA Methylation in Both Human and Animal Herpesvirus Infection

**DOI:** 10.1155/tbed/8542827

**Published:** 2025-07-31

**Authors:** Xiangqi Qiu, Jiajing Tian, Xuyang Zhao, Lucai Wang, Lele Wang, Yilin Bai, Aijun Sun, Guoqing Zhuang

**Affiliations:** ^1^International Joint Research Center of National Animal Immunology, The College of Veterinary Medicine, Henan Agricultural University, Zhengzhou 450046, Henan, China; ^2^School of Agricultural Sciences, Zhengzhou University, Zhengzhou 450001, Henan, China; ^3^Longhu Laboratory of Advanced Immunology, Zhengzhou 450046, Henan, China; ^4^Ministry of Education Key Laboratory for Animal Pathogens and Biosafety, Henan Agricultural University, Zhengzhou 450046, Henan, China

**Keywords:** gene expression, m^6^A methylation modification, molecular mechanism, vaccine, viral replication

## Abstract

During human herpesvirus infection, dynamic alterations of N6-methyladenosine (m^6^A) modification have been extensively observed in viral and cellular transcriptomes. This modification plays a crucial role in RNA metabolism, serving as a novel regulator of gene expression alongside DNA and protein modifications. Notably, reversible changes in a single m^6^A modification site can impact viral replication and pathogenicity. Recent studies have reported changes in m^6^A modification-associated epitranscriptomes and their functional analysis during animal herpesvirus infections. This review focuses on the research progress of m^6^A modification on the transcriptome in both human and animal herpesvirus infections within the same family. Specifically, it examines the dynamic alterations of m^6^A modification-associated epitranscriptomes, the expression of m^6^A-machinery proteins, regulatory molecular mechanisms associated with herpesvirus infection, and potential clinical applications. By addressing the gaps in research on m^6^A modification in animal viruses, new insights into the regulatory molecular mechanisms of viral diseases may be uncovered. Furthermore, natural hosts infected with animal herpesvirus serve as valuable biomedical models for studying the regulation of m^6^A modification on viral replication and pathogenesis, thereby supporting the development of novel vaccine and drug targets.

## 1. Introduction

The *Herpesviridae* family encompasses a diverse group of enveloped viruses characterized by large double-stranded DNA genomes, which are further categorized into alpha, beta, and gamma subfamilies [1]. Among the human prototypical herpesvirus are Herpes simplex virus type I (HSV-1), a member of the alpha-herpesvirus subgroup, which commonly causes skin and mucous membrane infections, as well as severe conditions like herpes simplex encephalitis [2]. Human cytomegalovirus (HCMV) belongs to the beta-herpesvirus category and is a prevalent virus known to cause birth defects and neurodevelopmental abnormalities in fetuses, with recurrent infections often proving fatal in transplant recipients [3]. Kaposi's sarcoma-associated herpesvirus (KSHV), also referred to as human herpesvirus type 8 (HHV-8), is a gamma-herpesvirus associated with diseases such as Kaposi's sarcoma (KS), primary exudative lymphoma (PEL), and multicentric Castleman's disease (MCD) [4]. Last, Epstein–Barr virus (EBV), designated as human herpesvirus type 4 (HHV-4), exhibits a unique capability to influence the differentiation of B lymphocytes into a distinct plasma cell-like state [5]. Current research has highlighted the involvement of N6-methyladenosine (m^6^A) modification in the infection processes of these herpesvirus [6, 7]. Nevertheless, conducting experiments outside the natural host environment, namely the human body, presents several limitations, such as variations in extracellular conditions, immune responses, tissue-specific effects, long-term consequences of infection, and responses to therapeutic interventions and drugs.

Pseudorabies virus (PRV) and Marek's disease virus (MDV) are animal alpha-herpesvirus that can serve as valuable models for understanding alphaherpesvirus-associated pathogenesis and gammaherpesvirus-associated oncogenesis [8, 9]. PRV infection leads to pseudorabies (PR), a highly pathogenic and infectious disease characterized by symptoms like high fever, itchiness, and encephalomyelitis, often resulting in mortality rates reaching up to 100%. PRV is known to infect not only its natural host, pigs, but also other mammals and rodents, including dogs, cats, and mice. Recent findings have shown PRV's ability to infect human beings [10]. On the other hand, Marek's disease (MD) caused by MDV is an immunosuppressive disease that triggers rapid tumor development in infected chickens [8]. The MDV life cycle in chickens encompasses four distinct infectious phases: the early cytolytic infection phase (which affects chickens' B-lymphocytes 3–6 days after inhalation of virus particles), the latency phase (emerging in activated CD4+ T-lymphocytes 7–10 days postinfection), the reactivation phase (stimulating late cytolytic infection), and the transformation phase (resulting in tumors across various organs in latently infected CD4+ T-lymphocytes) [11]. Despite the availability of vaccinations for MD, MDV continues to evolve to enhance its virulence, with the underlying molecular mechanisms not yet fully elucidated. Prior investigations have illustrated how various herpesvirus employ epigenetic modifications, such as DNA methylation and histone modification, to influence gene expression and replication. Recently, the emerging concept of epigenetic modifications on RNA has introduced a new dimension to the regulation of gene expression and viral replication [12].

RNA modifications are a widespread posttranscriptional regulatory mechanism, with more than 170 unique types identified across various RNA species, such as messenger RNA (mRNA), transfer RNA (tRNA), ribosomal RNA (rRNA), circular RNA (circRNA), and long noncoding RNA (lncRNA) [13]. These modifications play crucial roles in regulating RNA processing and translation by enhancing interactions with reader proteins and translation factors. In tRNA, modifications in the T and D loops influence tRNA folding and stability, while those in the anticodon loop impact codon recognition, decoding efficiency, and accuracy [14]. Conversely, rRNA modifications predominantly occur at functional ribosomal sites, such as the peptidyl transferase center and decoding site, affecting ribosomal biogenesis, structure, and function, consequently, regulating mRNA translation [15]. Methylation modifications like N6-methyladenosine (m^6^A) [16], N1-methyladenosine (m^1^A) [17], and 5-methylcytosine (m^5^C) [18] are extensively studied, each exerting specific functions in diverse biological contexts. The most prevalent mRNA modification in eukaryotes is m^6^A modification, with key regulatory proteins, including writers, readers, erasers, and suppressors [19]. The m^6^A modifications are highly conserved and are prevalent on various RNAs, predominantly near mRNA termination codons [20]. Upon surpassing a specific threshold, m^6^A-modified mRNA transcripts undergo proper splicing, translocation, translation, or degradation by reader proteins, participating in diverse physiological processes, including cap-dependent translation, RNA splicing, mRNA stabilization, and microRNA (miRNA) biogenesis. Disruption of these modifications can lead to imbalances in RNA metabolism, impacting various physiological and pathological processes, such as meiosis, biological clock regulation, DNA damage repair, stress response, circadian rhythms, cancer, and viral infections [12, 21–26].

High-throughput sequencing technologies have advanced considerably in the study of RNA modifications, leading to the gradual revelation of the widespread presence of m^6^A methylation marks in viral transcriptomes. This discovery suggests that m^6^A modifications play a significant role in viral replication and pathogenesis by influencing gene expression [27]. Notably, it has been confirmed that m^6^A modification contributes to the epitranscriptome alterations induced by various members of the *Herpesviridae* family [6, 12]. While several excellent reviews have discussed the regulatory role and molecular mechanisms of m^6^A modification in viral infections, this review focuses primarily on the latest research developments regarding m^6^A modification in human herpesvirus replication and pathogenicity. Additionally, we provide a summary of the most recent research progress on m^6^A modification in animal herpesvirus replication and pathogenicity, a topic explored for the first time. Furthermore, we discuss potential research directions that include investigating the role and molecular mechanisms of m^6^A modification in both human and animal herpesvirus replication and pathogenesis. These insights may illuminate new pathways for vaccine development and the design of antiviral drugs.

## 2. m^6^A-Machinery Proteins

**Figure 1:**
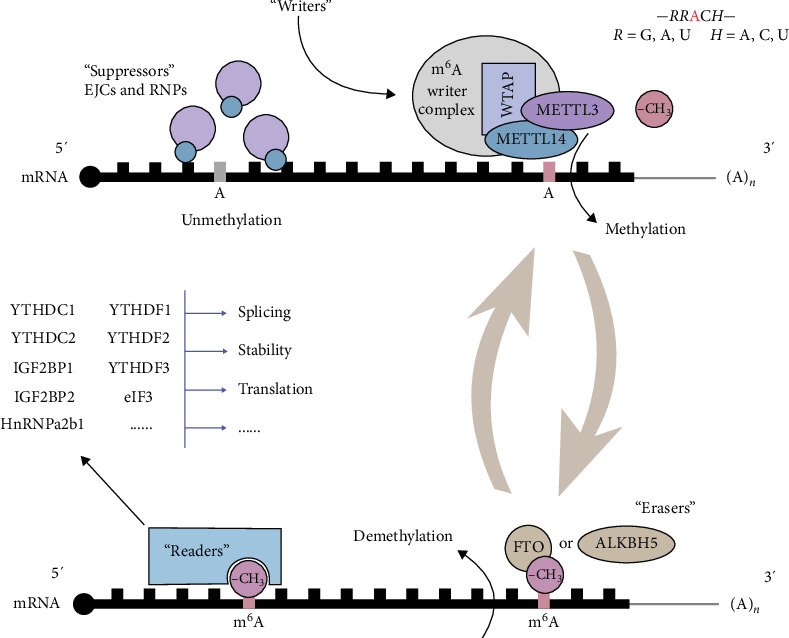
Dynamic regulatory mechanisms and biological functions of m^6^A modifications on mRNAs. In the context of the RRACH (R = A or G; H = A, C, or U) sequence, the m^6^A modification is catalyzed by the methyltransferase complex (MTC) comprising core subunits METTL3, METTL14, and WTAP, among others (writers). FTO and ALKBH5 function as “erasers,” enabling the removal of m^6^A modifications from mRNAs, thereby facilitating the reversibility of modifications. Reader proteins, such as YTHDF1-3, YTHDC1-2, and IGF2BP1-3, exhibit the ability to specifically recognize m^6^A modifications and mediate various biological functions of mRNAs, including splicing, stability regulation, translational efficiency, and degradation. Additionally, factors like EJCs can interfere with MTC access through spatial hindrance as suppressors, potentially inhibiting m^6^A formation and regulating its distribution and abundance on mRNA.

The regulatory mechanisms of m^6^A modifications can be classified into four main groups as follows: writers, erasers, readers, and suppressors. These groups collectively demonstrate a dynamic and reversible biological process in regulating RNA metabolism as described in Figure 1.

### 2.1. m^6^A Modification Writers

The m^6^A modification process is catalyzed by the m^6^A methyltransferase complex (MTC) (m^6^A modification writers), composed of core components, such as methyltransferase like 3 (METTL3), methyltransferase like 14 (METTL14), and various regulators, including Wilms' tumor 1-associating protein (WTAP), vir like m^6^A methyltransferase associated protein (VIRMA/KIAA1429), RNA binding motif protein 15/15B (RBM15/15B), Cbl proto-oncogene like 1 (HAKAI), zinc finger CCCH-type containing 13 (ZC3H13), and methyltransferase like 16 (METTL16) [28, 29]. METTL3, which binds to S-adenosylmethionine (SAM), serves as the central component of the MTC and is responsible for catalyzing the formation of RNA m^6^A modifications. Working in tandem, METTL14 forms a stable heterodimer with METTL3, aiding in the deposition of m^6^A on nuclear RNA in mammalian cells, and thereby heightening the catalytic capacity of METTL3. The combined action of METTL3 and METTL14 results in a significant enhancement of methyltransferase activity compared to their individual roles [28]. WTAP plays a crucial role by interacting with the METTL3-METTL14 complex to localize it to nuclear speckles. In the absence of WTAP, the RNA binding capability of METTL3 diminishes significantly [30]. Additionally, KIAA1429 (VIRMA) proteins interact with WTAP to facilitate the occurrence of m^6^A modifications near the 3′ untranslated region (UTR) and stop codons on mRNA [31]. The RBM15/15B complex recruits the WTAP-METTL3 complex to XIST, leading to the methylation of lncRNA-XIST [32]. ZC3H13 collaborates with the WTAP-VIRILIZER-HAKAI complex to regulate the m^6^A methylation modification levels of embryonic stem cells (ECs) [33]. Lastly, METTL16 participates in modulating SAM homeostasis formation and is involved in the m^6^A modification of nascent RNA in the nucleus [34].

### 2.2. m^6^A Modification Erasers

Two m^6^A demethylases, fat mass and obesity-associated protein (FTO) and ALK B homologue 5 (ALKBH5), have been identified as m^6^A modification erasers. These enzymes rely on divalent iron ions and 2-oxoglutarate for their enzymatic activity, enabling them to catalyze the demethylation of m^6^A-containing mRNA molecules. Generally, ALKBH5 and FTO are predominantly localized within the nucleus [35, 36]. FTO exhibits homology with the DNA repair protein AlkB and is implicated in the oxidative demethylation of 3-methylthymine within single-stranded DNA, as well as the demethylation of 3-methyluracil within single-stranded RNA [37]. Numerous investigations have highlighted the role of FTO as a proficient modulator of nuclear mRNA processing contributing to alternative splicing and the processing of the 3′ end of mRNA [38]. Furthermore, FTO is responsible for modulating the levels of N6, 2′-O-dimethyladenosine (m6Am), leading to selective regulation of m6Am-containing mRNA abundance in cells. Additionally, FTO plays a role in reducing the stability of m6Am mRNA, demonstrating a preference for demethylating m6Am over m^6^A [39]. ALKBH5 is involved in the removal of m^6^A modifications from nuclear RNA, particularly mRNA, both in experimental settings and in living organisms [36]. Moreover, ALKBH5 plays a significant role in the regulation of nuclear RNA export, metabolism, and gene expression, suggesting that reversible m^6^A modifications on RNA have extensive impacts on biological processes [40].

### 2.3. m^6^A Modification Readers

m^6^A-associated binding proteins, also known as m^6^A “readers,” play a key role in regulating gene expression by interacting with m^6^A sites on RNAs. This family of reader proteins (m^6^A modification readers) includes YTHDF1, YTHDF2, YTHDF3, YTHDC1, and YTHDC2. YTHDF1 functions by directly enhancing translation through binding to the m^6^A modification in the 3′UTR region [22], while YTHDF2 promotes mRNA decay by recruiting the CCR4-NOT deadenylation complex [41]. YTHDF3, acting as a cofactor for both YTHDF1 and YTHDF2, contributes to their regulatory functions [42]. YTHDC1, predominantly found in the nucleus, controls mRNA export from the nucleus to the cytoplasm, and supports the formation of exonic inclusion bodies [43, 44]. On the other hand, YTHDC2 modulates RNA translation by interacting with the 40–80S subunit in the cytoplasm [45]. Recently, Huang et al. [46] identified a new group of insulin-like growth factor two mRNA-binding proteins that exhibit a strong affinity for m^6^A-modified mRNAs via the canonical m^6^A motif GG (A)C, leading to increased mRNA stability. In addition to the YTH protein family, other proteins like eIF3 and HNRNP2AB1 have been recognized for their ability to recognize m^6^A modifications [47, 48]. Specifically, eIF3, a component of the 43S preinitiation complex, aids in protein translation by binding to the m^6^A site in the 5′UTR region of mRNA [47]. HnRNPA2/B1 directly binds to and regulates the processing of m^6^A-modified transcripts, including subset of primary miRNA transcripts by interacting with the miRNA microprocessor complex protein DGCR831 [48].

### 2.4. m^6^A Modification Suppressors

Exon junction complexes (EJCs) interact with mRNA sequences located upstream of exon junctions and are a prevalent constituent of messenger ribonucleoprotein (mRNP) complexes (m^6^A modification suppressors [49]. The core components of EJCs include three essential proteins: Eukaryotic Translation Initiation Factor 4A3 (EIF4A3), RNA Binding Motif Protein 8A (RBM8A), and Protein Mago Nashi Homolog (MAGOH) [50]. Initial studies suggested that EJCs might influence the nonuniform distribution of m^6^A modifications across the transcriptome by physically hindering them [51]. EJCs package mRNPs, making regions near exon junctions resistant to m^6^A modification and influencing the spatiotemporal distribution of these modifications along nascent RNA transcripts [52]. Through deep learning modeling, researchers found that the absence of pre-mRNA splicing in the host gene increases m^6^A modifications [53]. EJCs inhibit METTL3-mediated m^6^A modifications near splice sites within the coding sequence (CDS), leading to enhanced m^6^A enrichment in the 3′UTR and shaping the landscape of m^6^A modifications [54]. By acting as suppressors of m^6^A methylation, EJC proteins safeguard regions of transcripts lacking m^6^A modifications, guarding RNA proximal to CDS near exons and ultimately affecting mRNA stability through m^6^A modification regulation [49]. However, the role of EJCs in suppressing m^6^A at exon-intron boundaries is partial and limited to specific short internal exons [49].

## 3. Dynamic Regulation of the m^6^A Modification-Associated Epitranscriptome During Human Herpesvirus Infection

### 3.1. HSV-1

HSV-1 is a double-stranded DNA virus characterized by its genome structure, which comprises unique long (UL) and unique short (US) regions, flanked by terminal repeat (TR) and internal repeat (IRL) sequences [55]. Research conducted in 1977 first identified m^6^A modifications on HSV-1 mRNAs [56]. Recent advancements in m^6^A modification sequencing methods have led to the identification of at least 12 m^6^A modification peaks within HSV-1 transcripts, encompassing genes such as *UL1*, *UL2*, *UL12*, *UL28*, *UL29*, *UL38*, *UL39*, *UL42*, *UL46*, *UL49*, *US10*, *US11*, and *US12*. Following HSV-1 infection, an increase in the expression levels of the RNA methyltransferases METTL3 and METTL14, along with the YTHDF family of m^6^A readers (YTHDF1, YTHDF2, and YTHDF3), has been observed at the early stages of infection. Silencing of METTL3 has been shown to inhibit viral replication by downregulating the expression of both early genes (such as *ICP0*, *ICP8*, and *UL23*) and late genes (including *VP16*, *UL44*, *UL49*, and *ICP47*) [57]. Notably, during HSV-1 infection, METTL3 and METTL14 translocate from the nucleus to the cytoplasm, a process that is facilitated by the viral ICP27 protein [58]. Furthermore, after HSV-1 infection of human oral epithelial cells, a downregulation of the m^6^A demethylases ALKBH5 and FTO occurs, resulting in an increase in the overall m^6^A modification levels within these cells. Silencing of ALKBH5 and FTO has been implicated in the promotion of type I interferon (IFN-I) and interferon-stimulated genes (ISGs) expression, thereby enhancing the antiviral immune response and inhibiting viral replication [59]. Conversely, HSV-1 infection leads to the degradation of m^6^A-containing transcripts by YTHDF proteins, contributing to the downregulation of host m^6^A modifications. In particular, the knockdown of YTHDF proteins has been shown to diminish the expression of viral proteins while upregulating ISGs expression [60]. Notably, in the late stages of HSV-1 infection, the downregulation of YTHDF2 due to protein synthesis shutoff has been linked to an enhancement of the host's antiviral response. Additionally, METTL3 has been implicated in corneal neovascularization (CNV) during HSV-1 infection, regulated through canonical Wnt and VEGF signaling pathways in both in vitro and in vivo models. Specifically, METTL3 modulates the m^6^A levels of *LRP6* mRNA, thereby increasing its stability and protein expression, which fosters angiogenesis in HSV-1-infected human umbilical vein endothelial cells (HUVECs) [61]. Importantly, the m^6^A inhibitor 3-deazaadenosine (3-DAA) has been demonstrated to inhibit HSV-1 replication, suggesting a promising avenue for novel antiviral drug development [57].

m^6^A modification plays a multifaceted role in HSV-1 infection: it serves as both a tool for the virus to hijack host resources and a critical node in host defense. However, the aforementioned studies still leave many questions unresolved. For example, the nuclear-cytoplasmic transport of m^6^A modification enzymes (e.g., METTL3/METTL14) during early infection is mediated by ICP27, but the specific molecular mechanisms (such as whether phosphorylation or acetylation modifications are involved) remain unclear [62]; Addtionally, while HSV-1 infection causes YTHDF proteins to degrade host m^6^A-modified transcripts, it remains unknown whether the virus's own mRNA evade recognition through a similar mechanism. Futhermore, HSV-1 can establish latent infection in the trigeminal ganglion; does m^6^A modification participate in regulating latent-associated transcripts (such as LAT)? Do dynamic changes in m^6^A during viral reactivation influence the reactivation process? These questions remain to be explored in depth. Future research should focus on elucidating the spatiotemporal dynamics of regulatory mechanisms and developing precision treatment strategies targeting m^6^A to address the challenges posed by HSV-1 infection and its associated pathologies.

### 3.2. HCMV

HCMV, the prototypical beta-herpesvirus subfamily, has a genome of approximately 250 kb that encodes more than 200 proteins [63]. Within HCMV-encoded lncRNAs, m^6^A modifications have been identified, contributing to their stability and function through interactions with reader proteins [64]. In the context of HCMV infection, there is an upregulation of METTL3 and METTL14, along with increased expression of YTHDF1-3 and YTHDC1. Research has demonstrated that HCMV infection alters the m^6^A modifications of *IFNB1* mRNA. Specifically, depletion of METTL14 enhances the accumulation of *IFNB1* mRNA, leading to reduced HCMV replication, whereas depletion of ALKBH5 has the opposite effect [65]. Furthermore, HCMV infection has been shown to elevate the total m^6^A modification level in vascular endothelial cells. During HCMV infection, m^6^A modification has been linked to reduced stability and transcriptional termination of ubiquitin carboxy-terminal hydrolases *L1* (*UCHL1*) mRNA, contributing to inflammatory damage in the vascular endothelium [66]. METTL3-mediated m^6^A modification boosts the binding of mitochondrial calcium uniporter (MCU) mRNA to YTHDF3, leading to increased expression and facilitating HCMV-induced apoptosis in vascular endothelial cells. Notably, vitamin D3 downregulates the expression of METTL3 by inhibiting the activation of AMPK, thereby rescuing HCMV-induced apoptosis in vascular endothelial cells [67]. In summary, the alterations in m^6^A modifications induced by HCMV infection impact both viral and host cells, influencing viral infection strategies and virus–host interactions in a significant manner.

These findings suggest that HCMV exploits m^6^A modification as a double-edged sword that promotes viral replication and host damage. However, the aforementioned studies still leave several unresolved questions, including: (1) whether there are significant differences in the m^6^A modification profiles between the lytic and latent phases of HCMV; (2) whether the upregulation of METTL3/METTL14 and the YTHDF family by HCMV depends on virus-encoded proteins directly regulating the host epigenome; (3) whether m^6^A modification of HCMV's own RNA affects its stability, translation efficiency, or immunogenicity; (4) whether the virus utilizes the host m^6^A modification system to selectively regulate gene expression during distinct infection phases (lytic vs. latent); (5) whether HCMV's own RNA (e.g., the major immediate-early genes *IE1/IE2* that initiate viral replication) carries conserved m^6^A sites; (6) whether these modifications evade innate immunity by inhibiting RIG-I recognition (e.g., through masking the 5′ppp-m^6^A structure) to evade innate immunity. In-depth exploration of these questions is crucial for understanding the interaction between HCMV and its host.

### 3.3. KSHV

KSHV, a member of the gamma-herpesvirus subfamily, has a complex architectural structure in its mature virion. The virion comprises an envelope, a capsid protein layer, and a nucleocapsid enclosing almost 170 kb of double-stranded DNA. The viral genome exhibits distinct UL and US regions, each bordered by IR regions at the termini. These IR regions contain essential regulatory sequences and a subset of genes crucial for viral function. Within its genome, KSHV encodes around 80 to 90 open reading frames (ORFs) that are involved in various aspects of viral replication, immune evasion, and tumorigenesis. Despite this, the specific roles of the majority of ORFs in viral pathogenesis remain poorly understood, necessitating further investigation in the future to elucidate their functions in the overall course of the infection [68].

In the replication lifecycle of KSHV, abundant m^6^A modifications occur on its transcripts, particularly during the transition from latency to lytic phases [69]. These modifications are notably concentrated within the ORF regions, including *ORF46*, *ORF48*, *ORF49*, and *ORF50*. They play a crucial role in regulating viral gene expression, with the m^6^A site on *ORF50* being especially important [70]. This site enhances the stability of the *ORF50* transcript by recruiting *Staphylococcal nuclease* domain-containing protein 1 (SND1), which binds preferentially to m^6^A-tagged transcripts, particularly in their unspliced form. This interaction promotes early viral gene expression, influencing the viral replication cycle. Depletion of SND1 suppresses early KSHV gene expression, highlighting the significance of m^6^A modification in this context [71]. Further, investigations have shown that methylation induces a conformational shift in the RNA stem-loop structure within *ORF50*, transitioning it from a closed to a more open state at its apex. This methylation-induced structural change disrupts the energetically favored closed conformation, leading to altered stem-loop openness, which in turn affects transcript accessibility and stability [70]. Additionally, the m^6^A site on *ORF50* exerts regulatory control at the posttranscriptional level by recruiting YTHDC1, which facilitates splicing of the precursor mRNA, thereby regulating the expression of KSHV lethal genes [72]. Notably, the reader protein YTHDF2 can also bind to m^6^A-modified viral transcripts, modulating their stability [73]. These findings underscore the intricate regulatory mechanisms mediated by m^6^A modifications in KSHV replication and gene expression.

The latent phase of KSHV infection is characterized by dynamic reprograming of the host cellular epitranscriptome, resulting in a reduction of m^6^A modifications in the 5′UTR and an enhancement in the 3′UTR. These differential m^6^A modifications play a crucial role in cellular transformation processes, epithelial–mesenchymal transitions (EMTs), and various oncogenic pathways. These pathways include signaling cascades triggered by ephrin receptors, integrin-linked kinase (ILK), hypoxic conditions, bone morphogenetic proteins (BMPs), hepatic fibrosis, mammalian target of rapamycin (mTOR) activation, and adherens junction remodeling, which are essential for cancer-related EMT [73]. The transition of KSHV from latency to lytic replication leads to a reshaping of the m^6^A landscape on both viral and host mRNAs. Notably, the expression of G protein-coupled receptor class C group five member A (GPRC5A) is critical for KSHV's lytic replication, and m^6^A modifications are key in maintaining the stability of *GPRC5A* mRNA. This expression is induced by replication and transcription activator (RTA), a central regulator that coordinates the transition from latency to lytic replication in KSHV [74].

During KSHV infection, the *ORF37*-encoded SOX protein primarily degrades the majority of transcripts, although the *interleukin-6* (*IL-6*) transcript has the capability to evade immune-mediated degradation. This evasion is facilitated by the m^6^A modification on the *IL-6* transcript, preventing SOX-induced degradation by recruiting YTHDC2. Consequently, the protection and sustained expression of IL-6 are ensured [75]. METTL16 also plays a key role in KSHV lytic replication regulation, as demonstrated by the acceleration of KSHV lytic replication upon METTL16 knockdown, and its restraint upon overexpression. It has been observed that METTL16 influences the production of SAM by modulating the m^6^A modification of the transcript of a crucial enzyme in the SAM cycle, MAT2A. This modulation of the SAM cycle impacts KSHV lytic replication by regulating the balance of intracellular redox and levels of reactive oxygen species [76]. The m^6^A modifications yield diverse regulatory outcomes in specific cellular contexts, potentially enhancing or inhibiting KSHV gene expression. This variability stems from the specificity of m^6^A regulatory mechanisms across various cell types. The life cycle of KSHV is significantly influenced by the m^6^A pathway, with the functional consequences being dependent on the host cell type, highlighting the intricate interplay between the virus and host [77]. Furthermore, changes in m^6^A modification could potentially serve as biomarkers for diagnosing and monitoring KSHV infection. Further research is imperative to elucidate the precise mechanisms underlying m^6^A modification in KSHV infection, aiming to develop more effective prevention and control strategies.

However, the aforementioned studies still have many unresolved issues. the precise regulatory mechanism by which KSHV controls the activity and localization of the METTL3/METTL14 complex and demethylases (e.g., ALKBH5 and FTO) the reason why m^6^A modification of ORF50 promotes gene expression during early lytic stages but is actively cleared by viral nucleases (ORF37/SOX) in late stages—including whether this relates to the requirement for low-methylated RNA during viral packaging; the efficacy of existing m^6^A pathway inhibitors (e.g., 3-DAA) against KSHV; the potential of combining m^6^A regulators with immune checkpoint inhibitors (e.g., anti-PD-1) or oncolytic viruses; whether activating latent KSHV followed by m^6^A targeting could enhance the “activate and kill” strategy. These questions remain to be explored through further research.

### 3.4. EBV

EBV is a prototypical and common human tumor virus that can lead to the development of various lymphoid and epithelial cell carcinomas. The EBV genome is a linear double-stranded DNA genome approximately 170 to 180 kilobase pairs in length. It is structurally divided into UL and US regions, where most of the coding capacity for viral proteins is located. Flanking these regions are the IR domains situated at both ends of the genome, crucial for viral replication, and gene expression regulation. The EBV genome encodes around 80 to 90 ORFs [78]. However, the roles of the most EBV-encoded proteins remain under investigation, emphasizing the necessity for further research to comprehend their significance in viral biology and disease development.

During the lytic phase of EBV infection, the EBV protein BZLF1 interacts with the promoter of *METTL3*, leading to suppression of METTL3 expression, resulting in decreased m^6^A modification on *KLF4* mRNA. Consequently, the reduced m^6^A modification on *KLF4* transcripts evades degradation by YTHDF2, leading to upregulation of KLF4 protein expression, thus, promoting EBV infection in nasopharyngeal cells [79]. YTHDF1 contributes to inhibiting EBV replication through destabilizing the transcripts of *BZLF1* and *BRLF1* by recruiting RNA decay complexes ZAP, DDX17, and DCP2 [80]. Upon EBV infection, m^6^A modifications of cellular transcripts *DTX4* and *TYK2* are upregulated due to the downregulation of eraser protein ALKBH5 expression, resulting in diminished IFN production and enhanced viral replication [81]. YTHDFs proteins also suppress EBV replication via PIAS1-mediated SUMOylation. The cleavage of m^6^A pathway molecules by caspase leads to the stabilization of *BZLF1* transcripts, therefore, facilitating EBV replication [82]. These findings underscore the pivotal role of m^6^A modification in regulating EBV gene expression.

In the latent phase of EBV infection, the m^6^A modifications that emerged as a consequence of EBV infection were found to be distributed preferentially in the 3′UTR of cellular transcripts. Conversely, the modifications that were lost as a result of EBV infection were distributed preferentially in the CDS region of mRNAs. Notably, viral genes *EBNA2* and *BHRF1* undergo m^6^A modification. Knockdown of METTL3 results in the reduction of EBNA2 expression [83]. Additionally, EBV infection significantly upregulates METTL14, which subsequently, mediates m^6^A modification and upregulates the expression of the viral latent antigen EBNA3C [84]. It has been demonstrated that EBNA1 degrades METTL3 via the K48-linked ubiquitin–proteasome pathway, resulting in the downregulation of m^6^A modifications on *TRL9* mRNA. This downregulation, in turn, reduces TRL9 protein expression and facilitates immune evasion by the EBV [85].

METTL3 exerts a positive regulatory effect on pri-miR-BART3-3 p through its interaction with the microprocessor protein DGCR8 in an m^6^A-dependent manner, leading to a reduction in PLCG2 that promotes the proliferative capacity and tumor growth of NK/T cell lymphoma (NKTCL) cells in vitro and in vivo [86]. Furthermore, EBV-circRPMS1 has been shown to promote the progression of EBV-associated gastric cancer (EBVaGC) by recruiting Sam68 to the *METTL3* promoter, inducing METTL3 expression [87]. Knockdown of METTL3 or inhibition of methylation using 3-DAA and UZH1a resulted in decreased viability of EBV-positive tumor cells [88]. Additionally, EBV-circRPMS1 can regulate WTAP by affecting the NF-κB signaling pathway, which in turn impacts the proliferation and migration of gastric cancer cells [89]. YTHDF3 recruits DDX5 to inhibit IFITM1 expression, enhancing EphA2-mediated EBV entry into embryonic stem cells (ECs) [90]. FTO plays a critical inhibitory role in EBVaGC metastasis and invasiveness through an m^6^A-FOS-IGF2BP1/2-dependent mechanism [91]. METTL3′s high expression in NKTCL indicates poor prognosis [86]. M^6^A levels are crucial for diagnosing gastric cancer, demonstrating greater sensitivity and specificity than traditional tumor markers carcinoembryonic antigen (CEA) and carbohydrate antigen199 (CA199). Combining m^6^A with these markers can enhance diagnostic accuracy, making m^6^A an effective biomarker for diagnosing and monitoring gastric cancer [92]. These findings underscore the significance of m^6^A modification in the pathogenesis of EBV-associated malignancies. The potential of targeting m^6^A-associated modifying enzymes to treat EBV-associated cancers warrants further investigation.

During the lytic phase and latent phase of EBV infection, m^6^A modification exhibits distinct regulatory mechanisms. Lytic phase: the virus hijacks the m^6^A system to promote replication and immune evasion; latent phase: m^6^A remodels the host epigenetic landscape to sustain oncogenesis.

However, the above studies still leave several unresolved issues. the precise mechanism by which EBV regulates host m^6^A modification enzymes (e.g., METTL3 and ALKBH5) to transition between lytic and latent phases; are there virus-encoded “readers” or “writers” directly involved in regulation? whether virus-encoded “readers” or “writers” directly participate in this regulation; the phase-specificity of dynamic m^6^A changes on viral and host mRNAs during the viral life cycle; the potential roles of viral lncRNAs/miRNAs (in addition to circRNAs) in competitively binding or recruiting m^6^A enzymes; whether EBV has evolutionarily hijacked the host m^6^A system to adapt to immune pressures across different host cells. By addressing these questions, we may gain a more comprehensive understanding of the interaction between EBV and the host m^6^A system, providing a theoretical foundation for developing novel antiviral strategies and cancer diagnostic tools.

## 4. Dynamic Regulation of the m^6^A Modification-Associated Epitranscriptome During Animal Herpesvirus Infection

### 4.1. PRV

PRV is classified as a member of the *Herpesviridae* family and the *Alphaherpesvirinae* subfamily. The mature PRV particle is composed of an envelope, a layer of tegument proteins, a nuclear capsid, and a double-stranded DNA genome approximately 150 kilobases in length. This genome is organized into a UL region and a US region, both of which are flanked by inverted repeat (IR) regions. Within its genome, PRV contains over 70 ORFs and is estimated to encode between 70 and 100 proteins, the functions of many of which remain unknown [9].

PRV-infected swine testicle (ST) cells displayed a time-dependent decrease in total m^6^A levels, accompanied by a significant reduction in the expression of METTL3, METTL14, and WTAP. Further investigations revealed that during PRV infection, the viral US3 serine/threonine protein kinases phosphorylated METTL3, METTL14, and WTAP. Surprisingly, the viral US3 protein was observed to inactivate the m^6^A MTC in a kinase-independent manner, leading to the dissociation of the complex from chromatin [93]. During PRV infection, YTHDF readers were found to localize to P-bodies, where fewer but larger puncta could be observed in PRV-infected cells, leading to an increased degradation rate of mRNA. The number of P-bodies was also shown to increase during infection. Notably, YTHDF knockdown suppressed PRV protein production and led to an upregulation of IFN expression, suggesting that PRV's alteration of m^6^A modification serves as a viral immune evasion strategy [60]. Additionally, in a separate study, PRV-infected porcine kidney epithelial (PK15) cells exhibited abundant m^6^A modifications in viral transcripts. Knocking down the expression of writer proteins (METTL3 and METTL14) or reader proteins (YTHDF2 and YTHDF3) was found to inhibit PRV replication, whereas silencing the eraser protein ALKBH5 promoted replication [94].

However, the specific role and mechanisms of m^6^A modification during in vivo PRV infection remain unknown. Key unsolved questions include: whether PRV regulates viral gene silencing/reactivation via m^6^A modification when establishing latency in the peripheral nervous system; the functional significance of m^6^A during latent infection (current studies focus primarily on acute infection); the molecular mechanisms underlying US3-mediated inactivation of the METTL3/METTL14 complex, particularly its kinase-independent pathway; the role of m^6^A modification in PRV-infected pigs (in vivo validation is lacking as most studies use in vitro models); the therapeutic potential of targeting m^6^A-related enzymes (e.g., METTL3 inhibitors or ALKBH5 activators) against PRV. Resolving these questions will deepen our understanding of m^6^A modification in PRV infection and provide new targets for antiviral therapy.

### 4.2. MDV

MDV belongs to the family *Herpesviridae* and subfamily *Alphaherpesvirinae* based on the structure of its double-stranded DNA genome, which is about 180 kb in size [8]. The MDV genome exhibits a similar structure to that of PRV and HSV-1, encoding over 100 genes. Among these genes, those highly homologous and collinear with the genes of HSV-1 and Varicella-zoster virus (VZV) are located in the UL and US regions, while MDV-specific genes are found in the TR and IR regions [95]. There are three MDV serotypes: MDV-1 (Galid herpesvirus 2), MDV-2 (Galid herpesvirus 3), and MDV-3 (Meleagrid herpesvirus 1), with MDV-3 also known as the herpesvirus of Turkey (HVT). It is important to note that, while MDV-1 causes tumors in infected chickens, MDV-2 and HVT are nonpathogenic viruses. Currently, attenuated MDV-1, MDV-2, and HVT strains grown in cell culture can serve as vaccines against virulent virus infections; however, these vaccines are still insufficient in interfering with MDV replication. Hence, there is a need for new research to unravel the regulatory mechanisms underlying MDV pathogenesis and tumorigenesis comprehensively [96, 97].

During MDV replication in cell culture, we discovered a reprograming of m^6^A modifications on lncRNAs and circRNAs through the utilization of Methylated RNA immunoprecipitation sequencing (MeRIP-Seq) paired with bioinformatic analysis [98, 99]. Our findings revealed a close association between lncRNAs m^6^A modifications in MDV-infected chicken embryo fibroblasts and various signaling pathways including ErbB, GnRH, Toll-like receptor, Influenza A, and the MAPK pathway, all of which are linked to MDV infection [98]. Notably, during MDV infection, there was a noticeable reduction in the abundance of circRNAs m^6^A modifications. Subsequent analysis indicated a connection between the regulation of m^6^A modified circRNAs and the insulin signaling pathway [99]. In essence, the results imply that m^6^A modified lncRNAs and circRNAs exert significant regulatory roles in facilitating MDV replication in vitro.

In order to comprehensively understand the regulatory role of m^6^A modification during MDV infection [8], we initially leveraged the natural infectious disease model of MDV-infected chickens. Surprisingly, our investigation revealed that m^6^A modifications occurred on viral transcripts throughout the lytic infection, transformation, and reactivation phases. Subsequently, we delved into the alterations in the cellular m^6^A epitranscriptome across different stages of infection. Through MeRIP-Seq analysis, we identified dynamic changes in transcriptome m^6^A modifications over the MDV replication cycle in vivo, suggestive of MDV-driven reprograming of host transcriptional m^6^A modifications in an infection-cycle-dependent manner. Notably, our comparative analysis of cellular m^6^A modifications among the distinct infectious phases highlighted the extensive m^6^A modification of multiple immunity-associated transcripts. Prior research utilizing transcriptomic sequencing demonstrated that infection by highly virulent MDV leads to changes in the expression of immunity genes, potentially contributing to MDV immune evasion [100, 101]. However, further elucidation is required regarding the role and molecular mechanisms of m^6^A modification in regulating the expression of immunity-associated genes throughout the MDV life cycle. Moreover, our investigation into the expression of m^6^A modification-associated enzymes during MDV replication in chickens revealed altered expression profiles. Interestingly, the ectopic expression of the methyltransferase enzymes METTL3 or METTL14 was found to impact MDV replication, underscoring the pivotal regulatory role of m^6^A modifications in MDV replication and pathogenesis [11].

However, the aforementioned studies still have some unresolved issues. Why are METTL3/METTL14 suppressed during the lytic phase but highly expressed during the tumor phase? Is this differential expression mediated by specific viral proteins? How does m^6^A modification coordinate the promoting viral replication, while suppressing host immunity during the same infection stage? Is there selective regulation of virus RNA-specific methylation sites? During reactivation, are the dynamic changes in m^6^A modification associated with METTL3 nuclear translocation induced by host stress signals (such as the inflammatory factor IL-6)? Does MDV influence the activity of m^6^A modification enzymes by interfering with host metabolic pathways (such as itaconic acid or cholesterol synthesis)?

## 5. Conclusions and Future Perspectives

In this review, we summarize the latest research on m^6^A modifications during herpesvirus infections in humans (HSV-1, HCMV, KSHV, EBV) and animals (PRV, MDV), revealing its core role in virus–host interactions. Systematic analysis demonstrates that m^6^A functions as a “molecular switch” by dynamically reshaping the epitranscriptomic landscape of viral and host transcriptomes, thereby regulating viral replication cycles, immune evasion, and tumourigenesis.

Viruses hijack host m^6^A machinery to optimize gene expression: HSV-1 mediates the nuclear export of METTL3/14 via ICP27 to promote early gene expression [58]; KSHV utilizes m^6^A-SND1 interactions at ORF50 transcripts to enhance RNA stability, driving lytic reactivation [71]. m^6^A promotes immune evasion by: modulating viral gene immunogenicity (e.g., EBV BZLF1 inhibits METTL3 to evade TLR9 [85]); Reprograming immune signaling (e.g., TLR/MAPK remodeling in MDV infection [98]); suppressing antiviral pathways (e.g., HCMV downregulates IFNB1 via ALKBH5 [65]). m^6^A as a hub for carcinogenesis: latent-phase modifications drive oncogenesis by: stabilizing oncogene transcripts (EBV EBNA3C/EBNA2 [83, 84]); KSHV GPRC5A [74]); regulating noncoding RNAs (e.g., EBV-circRPMS1 [87]), forming “epitranscriptomic memory” to maintain malignancy.

It is worth noting that m^6^A regulation exhibits significant viral specificity and spatiotemporal dynamics: during the lytic phase, it promotes infection spread by enhancing the translation efficiency of viral transcripts or inhibiting host antiviral genes (e.g., HCMV *IFNB1* [65]); whereas during the latent phase, m^6^A reprograming drives cellular transformation by stabilizing oncogene transcripts (e.g., EBV *EBNA2* [84], KSHV *GPRC5A* [74]). In animal herpesvirus, the US3 protein of PRV inactivates the host MTC through a dual mechanism involving phosphorylation and kinase-independent mechanisms [93], while the dynamic changes in m^6^A modification during the MDV infection cycle are closely associated with host immune signaling pathways, such as TLR and MAPK [98].

However, existing research still has key limitations:

1. Current understanding of the mechanisms underlying m^6^A modification in herpesvirus infection primarily relies on in vitro models, with a lack of in vivo spatiotemporal resolution data on m^6^A dynamics during latent infection (e.g., HSV-1 in the trigeminal ganglion) and reactivation processes;
2. How virus-specific regulatory elements (e.g., EBV-circRPMS1, MDV lncRNA) modulate host modification networks through competitive binding or epigenetic memory remains unclear;
3. The post-translational modification of m^6^A enzyme activity (e.g., US3-mediated METTL3 phosphorylation) and its coupling mechanism with metabolic reprograming (succinic acid/SAM cycle) have not yet been elucidated.

To deepen our understanding of the role of m^6^A in herpesvirus infection, future research should focus on the following directions:

1. Exploration of virus–host interaction mechanisms: Identify how virus-encoded “hijacking factors” (e.g., EBV BZLF1, PRV US3) regulate the subcellular localization and activity of host m^6^A enzymes (METTL3, ALKBH5) through posttranslational modifications (phosphorylation/ubiquitination); elucidate how viral noncoding RNAs (e.g., KSHV miRNA, EBV lncRNA) modulate host gene exression via m^6^A modification/host pathways by competitive binding to m^6^A reading proteins (YTHDF2) or recruitment of modifying enzymes (e.g., METTL3).
2. High-precision modification profiling: Utilizing spatial transcriptomics combined with m^6^A-CLIP technology to elucidate the synergistic changes in three-dimensional genomic structure and modification sites during the viral “lysis-latent” transition; develop single-base resolution technologies (such as nanopore direct RNA sequencing or APOBEC-Cas13b-mediated site editing) to map the dynamic modification profiles of latent viral transcripts (e.g., HSV-1 LAT) and host non-coding RNAs (e.g., MDV circRNA).
3. Therapeutic strategies: Evaluate the synergistic efficacy of m^6^A inhibitors (3-DAA) combined with immune checkpoint inhibitors (anti-PD-1) or oncolytic viruses (e.g., HSV-1-derived T-VEC), and validate this synergy through humanized animal models (e.g., EBV-infected organoids).
4. Establish humanized animal models to simulate natural infection.

Studies on the regulatory mechanisms of m^6^A modification in herpesvirus infection not only reveal the fundamental principles of virus–host interactions but also provide new insights for vaccine and antiviral drug development. m^6^A modification plays a key role in viral replication cycles, immune evasion, and tumorigenesis by dynamically regulating the epigenetic landscape of the viral and host transcriptomes. Regulating m^6^A modification through inhibitors or agonists targeting enzymes involved in this process (such as methyltransferases and demethylases) offers a potential strategy for modulating viral replication. For example, 3-DAA is an inhibitor of SAH hydrolase [12] that blocks m^6^A modification of mRNA and exhibits broad-spectrum inhibitory effects against both DNA and RNA viruses. Its potential as an antiviral agent against animal viruses warrants further investigation. Additionally, the ability of m^6^A modification to influence mRNA expression provides a promising avenue for vaccine design targeting virus-specific mRNA sequences.

## Data Availability

The data sharing is not applicable to this article as no datasets were generated or analyzed during the current study.
